# Cancer-related fatigue in children during treatment: a 5-year cohort study of daily patient-reported outcomes with clinical implications

**DOI:** 10.1016/j.eclinm.2025.103607

**Published:** 2025-10-30

**Authors:** Alexander Tilg, Stefan Kuhle, Andreas Meryk, Benjamin Hetzer, Gabriele Kropshofer, Christina Salvador, Johannes G. Weiss, Jens Lehmann, David Riedl, Gerhard Rumpold, Alexandra Haid, Peter Willeit, Bernhard Holzner, Roman Crazzolara

**Affiliations:** a Department of Pediatrics, Medical University of Innsbruck, Innsbruck, Austria; b Institute of Clinical Epidemiology, Public Health, Health Economics, Medical Statistics and Informatics, Medical University of Innsbruck, Innsbruck, Austria; c Department of Psychiatry II, Medical University of Innsbruck, Innsbruck, Austria; d Ludwig Boltzmann Institute for Rehabilitation Research, Vienna, Austria; e Department of Psychiatry I, Medical University of Innsbruck, Innsbruck, Austria

**Keywords:** Cancer-related fatigue, Pediatric cancer, PROM, Supportive care

## Abstract

**Background:**

Cancer-related fatigue is one of the most common and distressing symptoms in pediatric oncology, yet its assessment and management remain insufficient. This study aimed to characterize trajectories of cancer-related fatigue in children and adolescents undergoing cancer treatment and to identify diagnosis- and therapy-specific fatigue predictors for personalized supportive care.

**Methods:**

In this prospective single-center cohort study, 104 pediatric cancer patients receiving chemotherapy (0–18 years) were enrolled at the Medical University of Innsbruck between May 1, 2020 and December 31, 2024. Daily symptom reports (patient or observer-reported) were collected via the ePROtect app and fatigue was scored 0–100, with lower values indicating greater burden. Patients were followed from diagnosis through the end of intensive therapy, and trajectories of cancer-related fatigue were analyzed across diagnostic groups and treatment phases using Locally Estimated Scatterplot Smoothing and linear mixed-effects models.

**Findings:**

In total, 11,602 daily cancer-related fatigue assessments were completed, with a median completion rate of 56·1%. Worst fatigue at diagnosis was seen in non-Hodgkin lymphoma (median: 54·2; interquartile range (IQR): 25·0, 75·0) and acute myeloid leukemia patients (median: 56·2; IQR: 33·3, 66·7); least in central nervous system tumor patients (median: 91·7; IQR: 75·0, 100). Glucocorticoid exposure was strongly associated with fatigue, especially in patients with acute lymphoblastic leukemia during steroid-intensive phases. Cancer-related fatigue was minimal during outpatient care but worsened during unplanned hospital admissions. Immunotherapy phases were associated with significant cancer-related fatigue improvement. Distinct fatigue patterns were observed across diagnoses and treatment protocols.

**Interpretation:**

Daily symptom monitoring reveals dynamic, diagnosis- and therapy-specific patterns of cancer-related fatigue in pediatric oncology patients. Glucocorticoid intensity and acute clinical events tend to be key drivers of fatigue burden. Integrating real-time symptom monitoring into clinical workflows helps identify high-risk periods for cancer-related fatigue and supports personalized supportive care to improve overall well-being in children and adolescents with cancer.

**Funding:**

Kinderkrebshilfe Tirol und Vorarlberg and Kinderhilfe Südtirol-Regenbogen supported this study.

**Research in context**

**Evidence before this study**

We conducted a PubMed based comprehensive literature review to identify existing studies published before August 31, 2025, focusing on cancer-related fatigue, its predictors, and symptom reporting in pediatric oncology. Studies were included, if they addressed “cancer-related fatigue,” “PROM in children and adolescents” “risk factors for cancer-related fatigue,” and their combinations. No language restrictions were applied. The literature indicates that research on the progression of cancer-related fatigue in children and adolescents throughout cancer therapy and its associated risk factors remains limited. Daily monitoring of cancer-related fatigue using patient-reported outcome measures (PROMs) has not yet been systematically explored in the pediatric oncology literature. Some studies suggest a correlation between cancer-related fatigue and chemotherapy, and preliminary data indicate that cancer-related fatigue levels may be more favorable in outpatient settings compared to inpatient environments.

**Added value of this study**

Our study uniquely captures daily patient-reported cancer-related fatigue across diverse pediatric cancer treatments, revealing dynamic, high-risk periods previously unrecognized. By identifying key factors influencing fatigue fluctuations, we provide actionable insights for personalized, real-time supportive care. This high-resolution data fills critical gaps left by prior infrequent assessments and small samples, advancing patient-centered management of cancer-related fatigue throughout therapy.

**Implications of all the available evidence**

Our findings demonstrate that daily PROM-based monitoring is both feasible and highly effective for identifying high-risk periods and guiding individualized supportive care in pediatric oncology. Integrating real-time PROM measurement into routine practice can transform symptom tracking into meaningful, patient-centered care. Future research should focus on implementing this approach across diverse clinical settings and evaluating its impact on patient outcomes.

## Introduction

Cancer-related fatigue is among the most prevalent and distressing symptoms in pediatric oncology, affecting around 70% of children and adolescents during and after cancer treatment, with profound impact on quality of life and functional capacity.^1^^,^^2^ As advances in treatment protocols have raised survival rates significantly, the long-term quality of survivorship – including comprehensive assessment and management of cancer-related fatigue – has become increasingly important and should be more integrated into routine clinical care.^2^

Recent longitudinal investigations demonstrate that fatigue experienced during treatment serves as powerful predictor of long-term outcomes. Children reporting fatigue during therapy face a 9- to 15-fold increased risk of persistent general fatigue in the first year post treatment, with 26% of acute lymphoblastic leukemia (ALL) survivors experiencing fatigue symptoms one year after end of treatment.^3^ Moreover, survivors of central nervous system (CNS) tumors exhibit more pronounced cognitive fatigue compared to those with solid tumors, with these differences persisting for at least five years after treatment.^4^ The International Late Effects of Childhood Cancer Guidelines Harmonization Group now recommends regular longitudinal fatigue surveillance using validated patient-reported outcome measures (PROMs) and effective interventions for fatigued survivors.^2^

Emerging evidence suggests that specific treatment modalities significantly influence fatigue trajectories.^3^^,^^5^^,^^6^ With the expanding application of immunotherapies, their association with fatigue is gaining increasing attention; initial evidence suggests, however, that certain agents, such as Blinatumomab, may be associated with less fatigue.^7^ In contrast, patients experiencing relapse, and consequently requiring more intensive treatment, appear to be at heightened risk of fatigue, although the specific therapeutic components underlying these associations remain poorly understood.^3^^,^^8^

To address these gaps, this study aims to systematically identify and quantify the treatment-related, clinical and demographic factors that are associated with fatigue development in pediatric cancer patients during therapy, utilizing a large-scale longitudinal dataset with comprehensive treatment characterization. This research enables unprecedented characterization of fatigue patterns and their associated factors. These findings will directly inform the development of two critical future outputs: (1) personalized intervention thresholds tailored to distinct clinical phases and risk profiles, and (2) practical implementation guidelines for integrating PROM-driven fatigue management into routine care.

## Methods

### Study design and partcicipants

This single-center cohort study conducted at the Division of Childhood Oncology, Medical University of Innsbruck (MUI), Austria, was ongoing from May 1, 2020, to December 31, 2024. It followed the Strengthening the Reporting of Observational Studies in Epidemiology (STROBE) reporting guidelines.^9^

We consecutively included all German-speaking children and adolescents aged from 0 to 18 years with cancer, who were receiving active treatment at our center. Exclusion criteria consisted of an expected treatment duration of less than 30 days at our department, the absence of chemotherapy, or the inability to complete the questionnaire due to physical or cognitive impairments. Patients who discontinued participation within the first 30 days or did not complete the specific fatigue questions were also excluded. All included patients were followed from the start of induction chemotherapy through all subsequent planned treatment phases up to the initiation of maintenance therapy or the final scheduled intravenous chemotherapy dose, and were monitored until two weeks after completion of therapy, death, or December 31, 2024, whichever occurred first.

### Ethics

The study received approval from the Ethics Committee of the MUI (EC number: 1055/2020).Written informed consent for data acquisition was obtained by clinical staff from all parents, together with assent from children 5 years and older.

### Procedures

All recruited patients or their primary caregivers were instructed to complete daily symptom reports, consisting of PROMs for patients old enough to self-report (years 5–18) or observer-reported outcome measures (ObsROMs) for patients 0–4 years via our ePROtect app. A detailed description of ePROtect and its use in daily clinical practice has previously been published.^10^^,^^11^

We collected PROMs or ObsROMs, sociodemographic data and treatment characteristics throughout active therapy. Cancer-related fatigue responses and levels were analyzed by treatment settings, as previously reported,^11^ including inpatient stay, unplanned admission, pediatric intensive care unit (PICU) and outpatient care referrals. Given the high heterogeneity of patient subgroups and their different treatment regimens, cancer-related fatigue scores and their association with treatment characteristics were analyzed in the more uniform group of lymphoproliferative diseases, where treatment approaches were relatively consistent, including acute lymphoblastic leukemia (ALL), acute myeloid leukemia (AML), Hodgkin's disease (HD), and non-Hodgkin's lymphoma (NHL). The ALL patients were stratified into a non-high risk and high-risk group, receiving treatment according to the Italian Pediatric Hematology Oncology Association (AIEOP) - Berlin-Frankfurt-Münster (BFM) ALL-2019 trial. AML patients were treated within AIEOP-BFM-AML 2020, HD within the German Pediatric Hematology Oncology Association - HD 2002 and NHL within NHL-BFM 2013 trials.

Depending on the diagnosis, the treatment phases included induction (with or without steroids), consolidation, reinduction (with or without steroids), Blinatumomab treatment phases, interim maintenance, other treatment phases, and periods without therapy. For statistical analysis, specific therapy elements were grouped based on their temporal relationship, drug properties, their side effect profiles, and our in-house clinical expertise to improve comparability and account for potential delayed effects on cancer-related fatigue. In the ALL non–high-risk group, induction and the first part of consolidation were combined due to their close temporal proximity. These were followed by the remaining part of consolidation, reinduction, and subsequently the Blinatumomab phase. Among ALL high-risk patients, induction and consolidation were also merged for similar reasons, followed sequentially by intensified consolidation — including, in specific patients, two phases of Blinatumomab — then reinduction and interim maintenance.

For AML treatment, the two separate induction blocks were followed by two consolidation periods, which were grouped together and concluded with a single reinduction phase. In HD patients, therapy elements were grouped into two induction periods followed by a combined consolidation phase. Similarly, in NHL, treatment phases consisted of one induction and one combined consolidation phase (Figure S2). Cancer-related fatigue values recorded during therapy breaks were attributed to the preceding treatment block, under the assumption of ongoing residual effects from prior therapy. To enhance visualization, individual treatment phases were displayed separately in the graphs.

### Measures

The daily symptom reports consisted of six concise questions in four domains: cancer-related fatigue, sleep, pain as well as nausea and appetite loss. Children aged 5–7 years answered using a three-face scale, while older children used a five-point Likert scale. For children under five, primary caregivers completed the questionnaires using the same five-point scale. The daily questionnaire was developed by a multidisciplinary group of clinicians and PROM researchers, following an eminence-based approach, as previously described.^11^ The need for very brief daily monitoring precluded the use of longer validated instruments such as the Pediatric Quality of Life Inventory. Cancer-related fatigue was assessed with two questions: (1) ‘Did you feel too tired yesterday to do anything?’ (based on National Comprehensive Cancer Network's recommendations^12^) and (2) ‘Was it difficult for you to move around yesterday?’ (added to capture daily life limitations related to physical fatigue). Scores were transformed to a 0–100 scale, and the final score was calculated as the mean of both question scores (5–7 y: 0/25/50/75/100; proxy/8–18 y: 0/12.5/25/37.5/50/62.5/75/87.5/100). Lower scores indicate greater symptom burden (i.e., more fatigue) and higher scores indicating lower burden.

### Outcomes

Our primary outcome was to assess the progression of cancer-related fatigue throughout therapy and its association with diagnostic and therapeutic factors.

### Statistics

Sample characteristics were summarized as counts and percentages or median and interquartile range (IQR) as appropriate. The completion rate was calculated by dividing the number of days on which symptom reports were completed by the number of days that each patient stayed in the study. Baseline cancer-related fatigue was calculated as the mean cancer-related fatigue over the first three days of therapy. To give each patient equal weight regardless of follow-up length, all cancer-related fatigue measurements within a patient were first averaged to yield a patient-level mean cancer-related fatigue score and the proportion of their observations in each severity category (none/mild 51–100, moderate 26–50, severe ≤25). Because the raw cancer-related fatigue scores are discrete, we used the mean to obtain a single continuous summary measure for each patient, from here on referred to as “patient-level cancer-related fatigue”. Across the cohort, patient-level cancer-related fatigue was then summarized overall and by patient characteristics (age, sex, diagnosis group) using the median and IQR, which are robust to non-normal distributions. To visualize the cancer-related fatigue trajectories over time, we applied Locally Estimated Scatterplot Smoothing (LOESS) within strata defined by treatment protocols in patients with lymphoproliferative tumors. For the formal modelling of cancer-related fatigue over time and across treatment blocks, we fitted linear mixed-effects models with treatment block as a fixed effect. The outcome was the median cancer-related fatigue per treatment block. To account for within-patient correlations across treatment blocks, we included a random intercept for each patient and specified a first-order autoregressive (AR (1)) correlation structure (Figure S3). Because some ALL high-risk patients received Blinatumomab instead of standard chemotherapy during the intensified consolidation block, we used a modified treatment-block variable that distinguished those groups. This indicator varies only between patients and therefore estimates the difference in median cancer-related fatigue in that block between patients who received Blinatumomab and those who received standard therapy. To assess changes in cancer-related fatigue over time, we re-specified the models iteratively with changing reference levels to estimate each treatment block's effect relative to the previous one. We also estimated the predicted median cancer-related fatigue for each treatment block along with 95% CIs. Although cancer-related fatigue scores are bounded between 0 and 100, we used a linear model for its interpretability and stability; while all predicted values remained within bounds, a small number of 95% CI upper limits slightly exceeded 100 and were truncated to reflect the scale of the outcome.

Missing data were handled using a complete case approach. To evaluate whether missing cancer-related fatigue data could bias the results, we conducted several sensitivity analyses. First, we compared completion rates across age, sex, and diagnosis group to assess whether missingness was associated with patient characteristics. Second, we examined the association between response rate and patient-level cancer-related fatigue using regression models, treating response rate both as a continuous variable and as a binary high/low indicator (completion rate at or above vs. below the cohort median), unadjusted and adjusted for age, sex, and diagnosis group. Finally, we examined three imputation scenarios: i) uniformly assigning moderately high cancer-related fatigue values (87·5) to all missing observations; ii) imputing missing values with random draws (with replacement) from each patient's observed cancer-related fatigue distribution; and iii) the same procedure as ii) but adding a modest upward shift of 12·5 to each sampled value to reflect a scenario where missing values were biased towards higher cancer-related fatigue.

The statistical analysis was performed using R version 4·5 and RStudio.^13^^,^^14^

### Role of the Funding source

The funders of the study had no role in study design, data collection, Formal analysis, data interpretation, or writing of the report.

## Results

Between May 1, 2020, and December 31, 2024, 143 children and adolescents diagnosed with cancer were screened for study eligibility, of whom 104 were enrolled (Figure S1, Table S1). The median age at enrollment was 7·1 years (IQR: 3·9, 12·3), and patients were followed for a median of 153 days (IQR: 85·0, 231·5). The most common diagnoses were lymphoproliferative diseases (66%), including acute lymphoblastic leukemia (40%). All patients received chemotherapy according to study criteria, and some additionally underwent surgery and/or radiotherapy. For a complete overview of patient characteristics, see Table 1.

**Table 1:** Demographic and clinical characteristics of the study cohort.

Characteristic	Patient data (N = 104)^a^
Age, median (IQR), y	7·1 (3·9, 12·3)
Age group	
0–4 y	34 (32·7)
5–7 y	23 (22·1)
8–18 y	47 (45·2)
Sex	
Male	62 (59·6)
Female	42 (40·4)
Family structure	
Single-Parent	20
Two-Parent	84
Siblings	
Yes	82
No	22
Surveillance, median (IQR), d	153 (85·0, 231·5)
Underlying diagnosis	
ALL	42 (40·4)
AML	8 (7·7)
HD	10 (9·6)
NHL	9 (8·7)
CNS tumor	10 (9·6)
Others	25 (24·0)
Treatment	
Chemotherapy	71 (68·3)
Chemotherapy plus surgery	18 (17·4)
Chemotherapy plus surgery plus radiotherapy	9 (8·7)
Chemotherapy plus radiotherapy	6 (5·8)

IQR, interquartile range; y, years; d, days; ALL, acute lymphoblastic leukemia; AML, acute myeloid leukemia; HD, Hodgkin disease; NHL, Non-Hodgkin lymphoma; CNS, central nervous system.

a Unless indicated otherwise, data are expressed as No. (%) of patients.

A total of 11,602 daily cancer-related fatigue assessments were completed over 21,371 person-days of surveillance, yielding a median completion rate of 56·1% (IQR: 30·3, 76·2). Completion rates did not differ by sex, age and diagnosis groups (Table S2) and showed no association with cancer-related fatigue scores (Table S3). Severe fatigue (score <25) was the most frequently reported symptom, occurring on 8·1% of assessed days, compared to lower frequencies for the other assessed symptoms pain (6·3%), sleep disturbances (4·5%), and nausea and appetite loss (3·4%).

Table 2 shows the patients-level's mean cancer-related fatigue scores around time of diagnosis and across treatment phases. At baseline, there were no significant differences in cancer-related fatigue levels between age and sex groups. Across diagnostic groups, patients with NHL and AML experienced worst cancer-related fatigue, with medians of 54·2 (IQR: 25·0, 75·0) and 56·2 (IQR: 33·3, 66·7), respectively. Also, cancer-related fatigue levels in patients with ALL high-risk differed from those in ALL non-high-risk, at 75·0 (IQR: 37·5, 91·7) and 83·3 (IQR: 50·0, 100), respectively. CNS tumor patients had least cancer-related fatigue with a score of 91·7 (IQR: 75·0, 100). Compared to baseline (75·0; IQR: 42·7, 91·7), overall cancer-related fatigue levels improved across treatment phases to 84·2 (IQR: 67·0, 94·4). The greatest improvement was noted in patients aged 5–7 years, as well as in those diagnosed with NHL, AML and ALL non-high-risk.

**Table 2:** CRF at baseline and across treatment phases.

Characteristic	CRF^a^ at baseline^b^	CRF^a^ across treatment phases
Overall (N = 104)	75·0 (42·7, 91·7)	84·2 (67·0, 94·4)
Age group		
0–4 y	75·0 (39·6, 89·6)	84·2 (71·9, 93·4)
5–7 y	75·0 (45·8, 100)	90·2 (72·2, 97·0)
8–18 y	75·0 (49·0, 87·5)	77·9 (58·9, 90·3)
Sex		
Male	75·0 (41·7, 95·8)	83·1 (66·2, 94·3)
Female	75·0 (45·8, 91·7)	87·3 (69·7, 94·7)
Underlying diagnosis		
ALL HR	75·0 (37·5, 91·7)	71·9 (63·8, 82·0)
ALL non-HR	83·3 (50·0, 100)	88·9 (77·9, 94·4)
AML	56·2 (33·3, 66·7)	65·5 (55·9, 80·6)
HD	79·2 (71·9, 85·9)	88·9 (77·5, 96·1)
NHL	54·2 (25·0, 75·0)	83·1 (69·6, 90·2)
CNS tumor	91·7 (75·0, 100)	95·0 (77·2, 95·9)
Others	77·1 (37·5, 87·5)	83·1 (60·8, 90·0)

CRF, Cancer-related fatigue; y, years; ALL, acute lymphoblastic leukemia; HR, high risk; AML, acute myeloid leukemia; HD, Hodgkin disease; NHL, Non-Hodgkin lymphoma; CNS, central nervous system.

a Each patient's CRF score was first summarized as the mean of all their individual CRF scores for the specified period (baseline or entire study). These patient-level means were then summarized across patients using the median and interquartile range (IQR).

b Baseline is defined as the first three days after diagnosis.

Outpatient care was associated with better fatigue scores compared to inpatient treatment (Table 3). The worst scores were observed during unplanned hospitalizations (69·9; 95% CI: 65·3, 74·4) and during PICU admissions (48·7; 95% CI: 42·0, 55·4).

**Table 3:** CRF by patient location.

Care setting	CRF^a^
Inpatient	
Planned admission	75·4 (71·4, 79·5)
Unplanned hospitalization	69·9 (65·3, 74·4)
PICU	48·7 (42·0, 55·4)
Outpatient	
Home	82·3 (78·1, 86·4)
Ambulatory care	82·4 (78·3, 86·6)

CRF, Cancer-related fatigue; PICU, pediatric intensive care unit.

a Patient-level mean fatigue scores were calculated for each location and analyzed with a linear mixed-effects model including a random intercept for patient. Model-based estimates and 95% confidence intervals represent the mean fatigue score for each location.

**Fig. 1:**
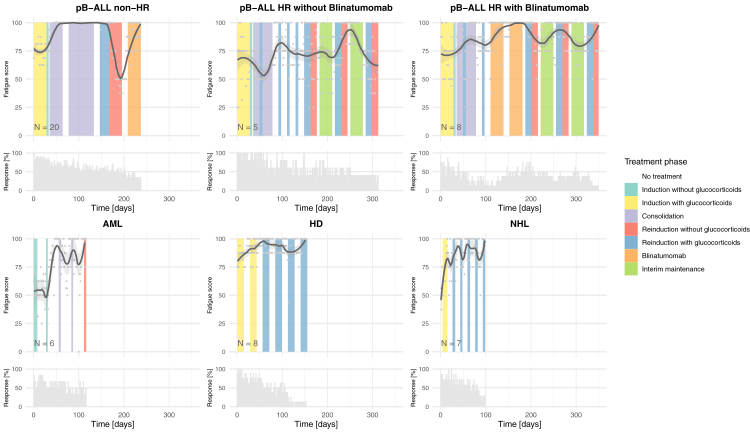
Progression of cancer-related fatigue throughout therapy modelled with LOESS smoothing. The progression of cancer-related fatigue is displayed throughout active therapy in lymphoproliferative diseases across six different treatment regimens. Higher scores indicate a lower symptom burden, while lower scores reflect greater fatigue. Therapy phases are color-coded according to the respective treatment regimens, based on protocol and clinical experience. Below each cancer-related fatigue progression visualization, the daily response rate of the patients (relative to all patients in that group) is provided. Among non-high-risk ALL patients, three out of 20 received Blinatumomab. In the case of HD patients, five out of eight received two instead of four reinductions. For NHL patients, six received three reinductions, and one received five. LOESS, Locally Estimated Scatterplot Smoothing; pB, pre B-cell; ALL, acute lymphoblastic leukemia; HR, high risk; AML, acute myeloid leukemia; HD, Hodgkin disease; NHL, Non-Hodgkin lymphoma.

Comprehensive longitudinal analysis of cancer-related fatigue trajectories across six treatment protocols revealed distinct temporal patterns shaped by diagnosis-specific therapeutic approaches and pharmacological interventions. Utilizing LOESS smoothing to characterize daily symptom burden, the data demonstrated that cancer-related fatigue severity was predominantly associated with glucocorticoid administration phases, though with notable variations across malignancies (Fig. 1, Table 4).

**Table 4:** Progression of CRF across treatment phases.

Diagnosis	Treatment phase	Δ CRF (95% CI)^a^	CRF (95% CI)^d^
**ALL non-HR (N = 20)**	Induction/Consolidation	Ref.	85·3 (76·5, 94·1)
	Consolidation	+9·1 (0·4, 17·7)	94·4 (85·6, 100)
	Reinduction	−19·9 (−29·3, −10·4)	74·7 (65·7, 83·7)
	Blinatumomab^b^	+24·9 (5·7, 44·1)	98·8 (82·4, 100)
**ALL HR (N = 13)**	Induction/Consolidation	Ref.	69·7 (59·8, 79·6)
	Intensified Consolidation (w/o B.)^c^	+6·9 (−8·2, 22·1)	76·7 (62·7, 90·7)
	Intensified Consolidation (w/B.)^c^	+19·0 (5·6, 32·5)	88·8 (76·5, 100)
	Reinduction I (w/o prior B.)	−3·4 (−19·6, 12·8)	73·3 (61·6, 85·0)
	Reinduction I (w/prior B.)	−15·5 (−30·5, −0·5)	73·3 (61·6, 85·0)
	Interim Maintenance I	+20·3 (5·6, 35·0)	93·6 (81·5, 100)
	Reinduction II	−19·3 (−34·5, −4·1)	74·3 (62·1, 86·5)
	Interim Maintenance II	+20·9 (5·1, 36·7)	95·2 (82·4, 100)
	Reinduction III	−33·5 (−49·8, −17·2)	61·7 (48·9, 74·5)
**AML (N = 6)**	Induction I	Ref.	59·1 (35·7, 82·5)
	Induction II	+27·8 (−3·6, 59·3)	87·0 (61·5, 100)
	Consolidation	+0·2 (−19·2, 19·6)	87·7 (62·1, 100)
	Reinduction	+18·8 (−15·3, 52·9)	92·8 (63·8, 100)
**HD (N = 8)**	Induction I	Ref.	82·8 (75·6, 90·0)
	Induction II	+10·9 (3·0, 18·9)	93·7 (86·5, 100)
	Reinduction	+0·3 (−5·8, 6·4)	94·2 (86·7, 100)
**NHL (N = 7)**	Induction	Ref.	82·1 (70·3, 93·9)
	Consolidation	+9·8 (−0·1, 19·8)	92·0 (80·2, 100)

CRF, Cancer-related fatigue; CI, confidence interval; ALL, acute lymphoblastic leukemia; w/o, without; w/, with; B., Blinatumomab; AML, acute myeloid leukemia; HD, Hodgkin disease; NHL, Non-Hodgkin lymphoma; HR, high-risk; Ref., referent.

a Coefficients are from linear mixed-effects models for each diagnosis with patient as a random effect and a first-order autoregressive correlation structure. Values reflect differences relative to the preceding treatment block and were obtained from re-specified models with changing reference levels. Predicted values (rightmost column) are from a single model with the first block as reference; minor discrepancies between modelled and calculated differences are expected due to re-parameterization.

b 3 out of 20 patients received Blinatumomab.

c 8 patients received Blinatumomab during Intensified Consolidation; 5 patients only received chemotherapy.

d Predicted values (median) based on the fixed-effects component of the model; confidence intervals were calculated using the model's variance-covariance matrix and the delta method.

In ALL, risk stratification and treatment intensity critically modulated cancer-related fatigue dynamics. ALL high-risk patients exhibited worse cancer-related fatigue scores during induction/consolidation compared to non-high-risk cohorts (69·7; 95% CI: 59·8, 79·6 vs. 85·3; 95% CI: 76·5, 94·1). In non-high-risk ALL, fatigue markedly deteriorated during steroid-intensive reinduction phases (Δ −19·9 at days 120–150, 95% CI: −29·3, −10·4). In contrast, fatigue improved during steroid-free consolidation periods (post-day 30) and approached near-resolution during Blinatumomab treatment (Δ +24·9 at days 120–180, 95% CI: 5·7, 44·1), in sharp contrast to conventional chemotherapy phases. This divergence was particularly evident when comparing ALL high-risk patients who received three blocks of chemotherapy containing high dose dexamethasone, with those who received only one block of chemotherapy followed by two blocks of Blinatumomab during intensified consolidation. Patients receiving immunotherapy exhibited a significantly greater improvement in cancer-related fatigue scores (Δ +19·0, 95% CI: 5·6, 32·5) compared to those receiving only conventional chemotherapy (Δ +6·9, 95% CI: −8·2, 22·1).

AML trajectories showed a distinct pattern, with peak fatigue (59·1; 95% CI: 35·7, 82·5) during initial induction followed by progressive improvement in subsequent cycles. Cancer-related fatigue resolution aligned closely with shorter induction/consolidation phases in later phases (Δ +27·8, 95% CI: −3·6, 59·3), stabilizing post-induction. Similarly, NHL regimens demonstrated stable cancer-related fatigue profiles with minimal fluctuation, likely attributable to limited cumulative steroid dosing.

In contrast, HD patients experienced low cancer-related fatigue burden (82·8; 95% CI: 75·6, 90·0) following induction, persisting throughout treatment despite protocol-specified steroid tapering (Figure S4).

Sensitivity analyses imputing missing cancer-related fatigue scores using three different approaches (assigning all missing values score of 87 5, sampling from each patient's observed values, or sampling plus an additional 12 5) showed trajectories that were visually similar to the primary analysis (Figure S5).

## Discussion

This study is the first to systematically capture daily PROMs and ObsROMs for cancer-related fatigue across the entire course of therapy in a diverse pediatric oncology cohort, using a longitudinal design that spans diagnosis and treatment. Such high-frequency real-world data provide new insights into the temporal dynamics of fatigue, its association with specific therapies, and the critical periods when specific interventions may be most beneficial.

Our findings reveal pronounced differences in baseline cancer-related fatigue levels across diagnostic groups, with the worst scores observed in children with NHL and AML. This likely reflects the distinct pathophysiology of these malignancies: NHL is often characterized by diffuse organ infiltration, including effusions into the pleura and peritoneal cavity, which can lead to significant discomfort and functional impairment even before therapy begins.^15^ Similarly, lymphoproliferative diseases such as AML and ALL frequently present with extensive bone marrow infiltration, resulting in pain, profound weakness, and reduced physical capacity.^16^^,^^17^ These disease-specific factors set a lower baseline for fatigue, which then evolves as treatment progresses. Encouragingly, cancer-related fatigue levels generally improve as remission is achieved, and disease burden recedes. However, this improvement is neither linear nor uniform; periods of deterioration are common, particularly during reinduction phases marked by increased treatment toxicity. Unplanned hospitalizations often triggered by complications such as mucositis and febrile neutropenia are closely associated with sharp declines in reported energy and well-being, highlighting the interplay between acute medical events and symptom burden.^12^^,^^18^

The utility of daily PROM-based monitoring becomes especially apparent when comparing our results to previous work. Recent studies by Dupuis and colleagues have demonstrated the benefit of routine symptom screening in pediatric oncology, showing that regular use of tools like SSPedi can improve symptom documentation and management.^19^^,^^20^ However, these studies were limited to short observation periods and did not capture the full therapeutic journey or the nuanced, diagnosis- and phase-specific evolution of fatigue. Our approach, with its high-frequency, long-term data collection, reveals that fatigue is not a static or isolated symptom but rather a dynamic marker of both disease activity and treatment effects. While routine screening in Dupuis’ work led to general improvements in symptom burden, our data show that the timing and severity of fatigue are closely associated with the underlying malignancy and the specific structure of treatment regimens. This level of detail enables the identification of high-risk periods and patient subgroups who may benefit most from targeted supportive care interventions, such as ALL patients during glucocorticoid exposure or those experiencing unplanned hospitalizations. Interventions can be tailored to these risk windows and may include structured exercise or relaxation techniques to reduce fatigue.

A particularly salient finding of our study is the central role of glucocorticoids in shaping fatigue trajectories. Steroid administration, especially during reinduction phases, consistently precipitated marked increases in fatigue severity across lymphoproliferative malignancies.^5^^,^^21^ This effect was most pronounced in ALL non-high-risk and high-risk patients, who experienced extended courses of high-dose steroids, but was also evident in other diagnoses. The mechanisms underlying steroid-induced fatigue are likely multifactorial, involving sleep disturbance, mood alterations, inflammation, and neuroendocrine disruption.^22^, ^23^, ^24^ Notably, our data also highlight the potential for therapeutic innovation: when glucocorticoid-intensive phases were replaced by immunotherapy - such as with Blinatumomab - patients experienced a substantial and sustained improvement in cancer-related fatigue. This observation aligns with emerging evidence that targeted therapies may offer not only superior disease control but also a more favorable symptom profile, reinforcing the importance of integrating PROMs into therapeutic decision-making as the field moves toward more personalized cancer care.^25^^,^^26^

The strengths of this study lie in its comprehensive, longitudinal design, high frequency of data collection, and inclusion of a broad range of diagnoses and treatment regimens. Nevertheless, several limitations must be acknowledged. As a single-center study, our findings may not be fully generalizable to other settings with different patient populations, treatment protocols, or supportive care resources. The observational nature of the study and the small sample size preclude conclusions about causality between specific interventions and changes in fatigue. Although fatigue is a multidimensional construct encompassing cognitive, emotional, and physical domains, our assessment was limited to the physical dimension, using a brief daily questionnaire that also captured several other bothersome symptoms. Additionally, while daily symptom reports provide rich and actionable data, their implementation in routine practice requires careful attention to workflow integration, clinician engagement, and patient or family burden.^27^^,^^28^ Despite these challenges, our experience demonstrates that sustained, high-frequency symptom monitoring can yield valuable insights that inform both clinical care and future research.^11^^,^^29^^,^^30^

For clinical practice, integrating real-time PROMs could help flag high-risk phases and enable interventions such as structured exercise or relaxation techniques, to reduce fatigue. For future research, these findings support multi-center validation studies and the development of age-specific supportive care strategies to optimize patient outcomes.

In conclusion, this study characterizes the trajectories and predictors of cancer-related fatigue in pediatric oncology, providing key insights into how fatigue evolves across diagnoses and treatment phases. Drivers such as glucocorticoid exposure and acute events were identified as critical periods of heightened risk, highlighting opportunities for personalized interventions to help patients manage their treatment burden. By capturing these complex patterns through daily symptom monitoring, clinicians can better anticipate high-risk phases and deliver timely, tailored supportive care, ultimately enhancing overall well-being and potentially reducing acute care utilization. As new therapies continue to reshape the landscape of childhood cancer treatment, the integration of real-time patient-reported data into clinical practice will be essential for ensuring that advances in survival are matched by improvements in the lived experience of young patients and their families.

## Contributors

Drs Tilg, Meryk and Crazzolara had full access to all the data in the study, verified the data, take responsibility for the integrity of the data and for the decision to submit the manuscript.

Drs Tilg, Crazzolara and Kuhle take responsibility for the accuracy of the data analysis.

Concept and design: Tilg, Meryk, Kuhle, Willeit, Crazzolara.

Acquisition, analysis, or interpretation of data: Tilg, Kuhle, Meryk, Kropshofer, Hetzer, Salvador, Weiss, Riedl, Lehmann, Haid, Willeit, Holzner, Crazzolara.

Drafting of the manuscript: Tilg, Meryk, Kuhle, and Crazzolara.

Critical revision of the manuscript for important intellectual content: Kropshofer, Hetzer, Salvador, Weiss, Riedl, Lehmann, Haid, Willeit, Holzner.

Statistical analysis: Kuhle.

Obtained funding: Crazzolara.

Administrative, technical, or material support: Rumpold, Riedl, Lehmann, Holzner, Crazzolara.

Supervision: Willeit, Meryk, Crazzolara.

All authors read and approved the final version of the manuscript.

## Data sharing statement

All data collected and analyzed during this study will be made available without time limit, upon reasonable request, to the journal editor or any person with responsibilities relevant to the publication process, in case additional information is required for verification or editorial review.

## Declaration of generative AI and AI-assisted technologies in the writing process

During the preparation of this work the authors used ChatGPT (OpenAI, 2025) in order to improve language and readability. After using this tool/service, the authors reviewed and edited the content as needed and take full responsibility for the content of the publication.

## Declaration of interests

Rumpold and Prof Holzner reported holding intellectual property rights to the software tool CHES. Lehmann reports consultancy for Evaluation Software Development and a research grant from Takeda, both outside of the submitted work. Riedl received grants from EORTC Qualitiy of Life Group and EU Horizon, both outside the submitted work. Prof Willeit received consulting fees of Novartis Pharmaceuticals without association to this work. No other disclosures were reported.
