# Supplementary figures and images for: Cancer-related fatigue in children during treatment: a 5-year cohort study of daily patient-reported outcomes with clinical implications
Source: eClinicalMedicine. 2025 Oct 30;90:103607. doi: 10.1016/j.eclinm.2025.103607 (PMC12613073; doi:10.1016/j.eclinm.2025.103607)

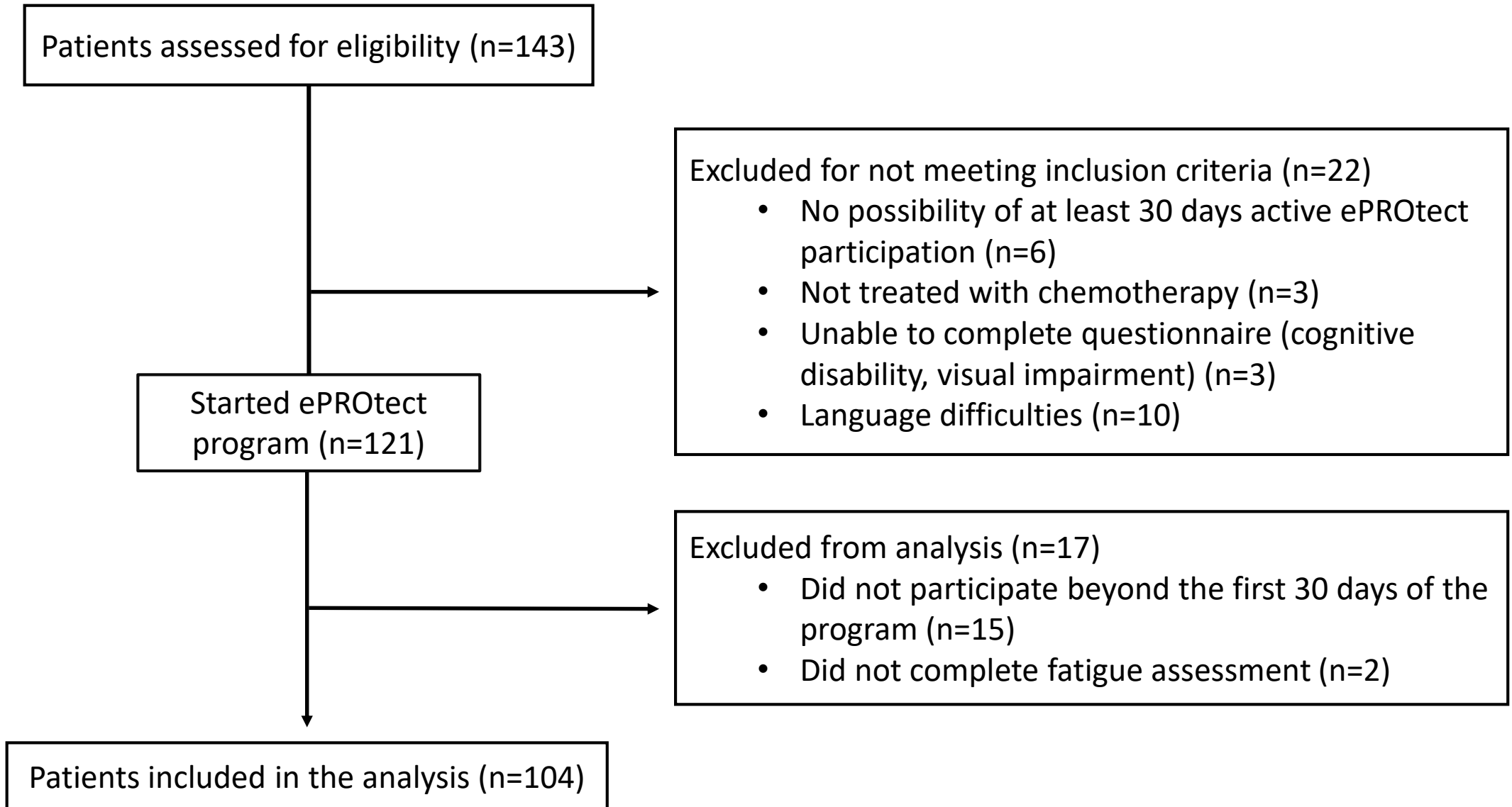

Supplement: Figure S1 [file mmc1.pdf]

Predicted Median Fatigue Score

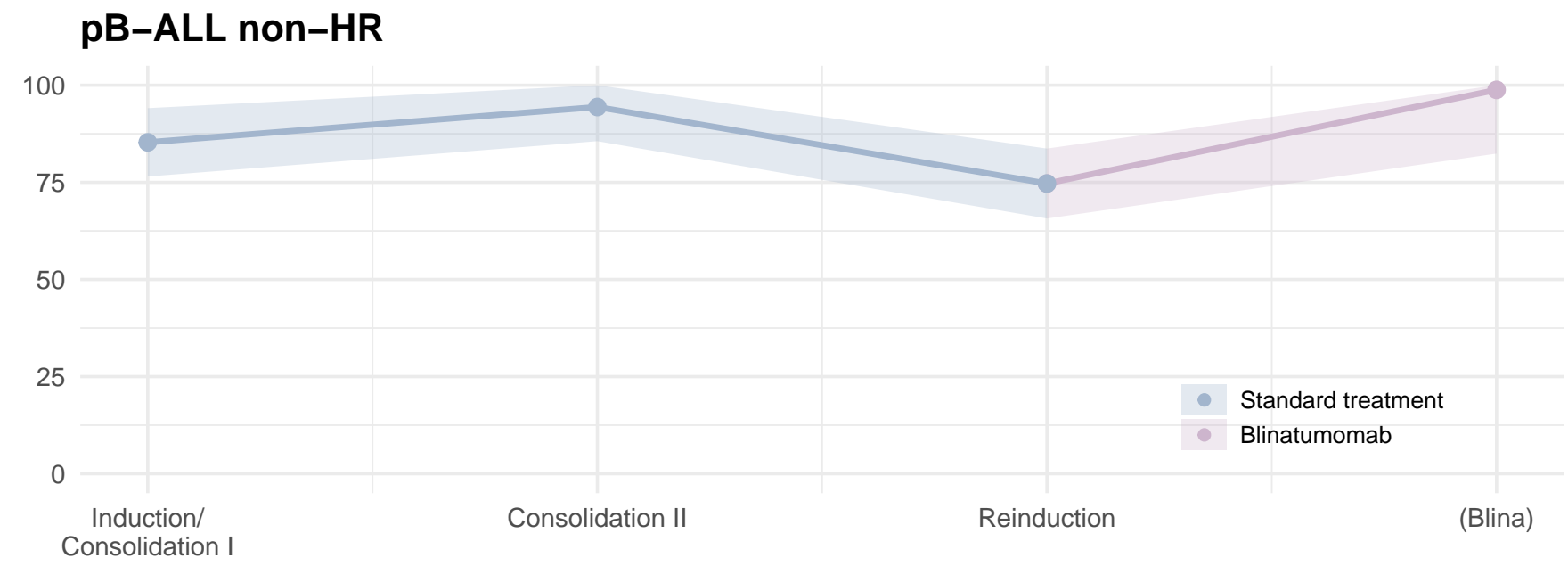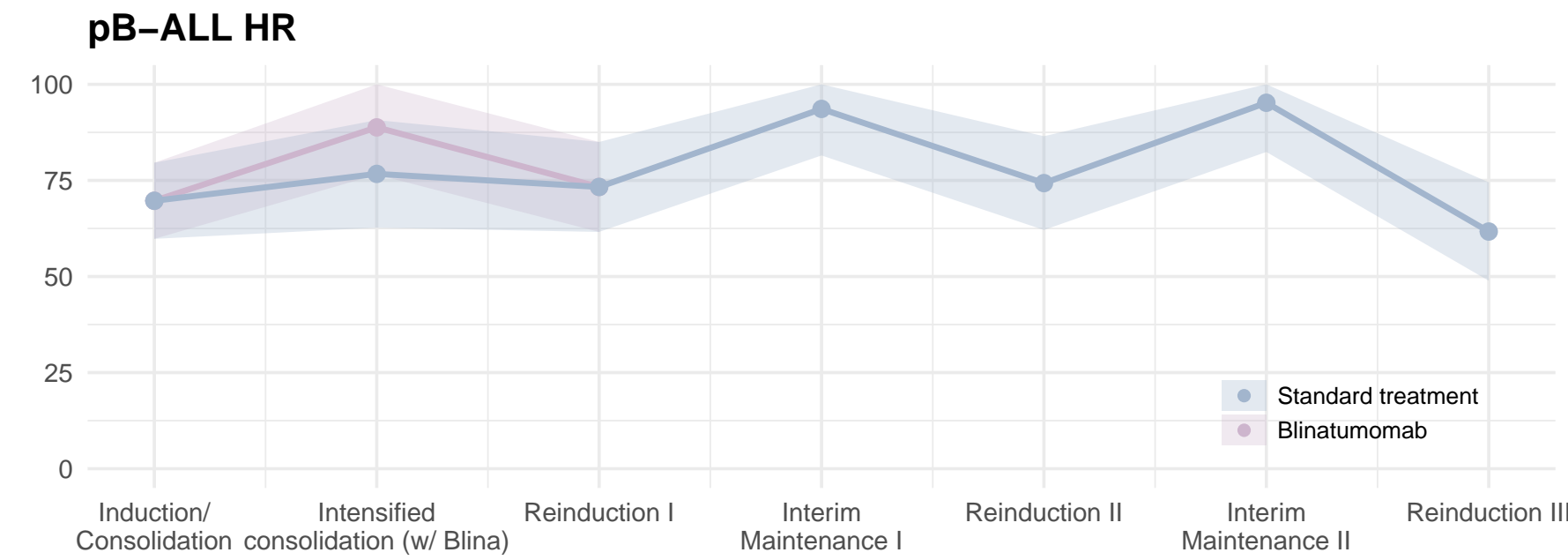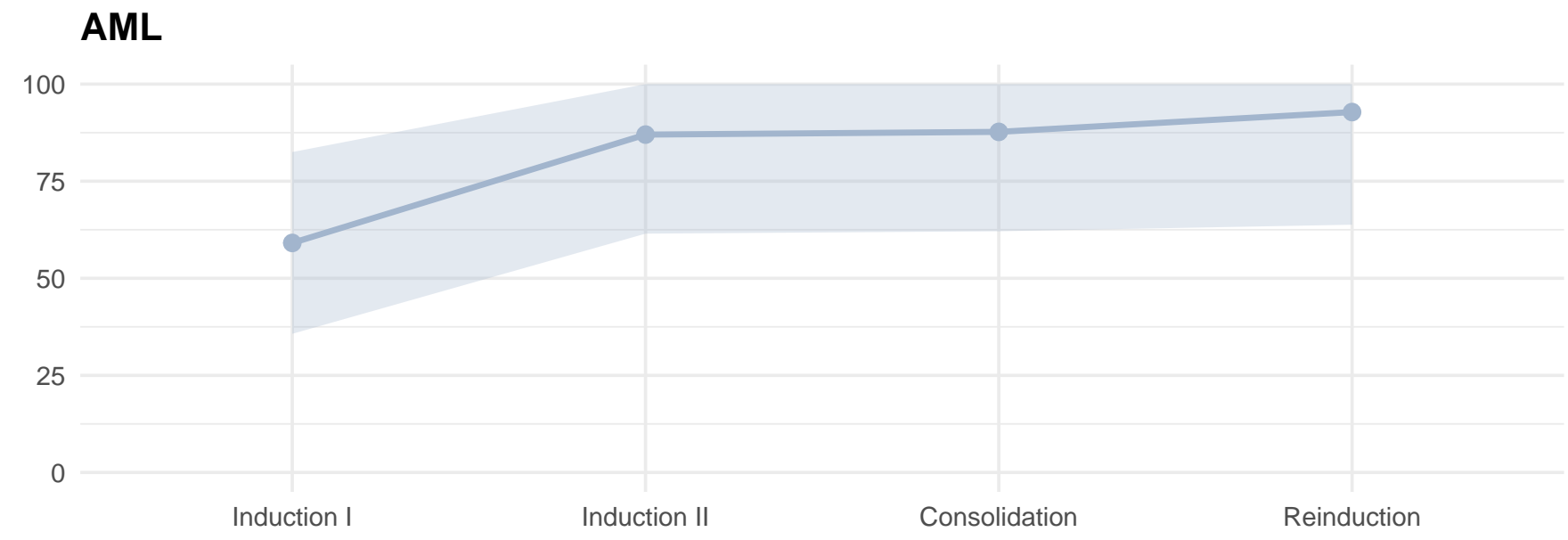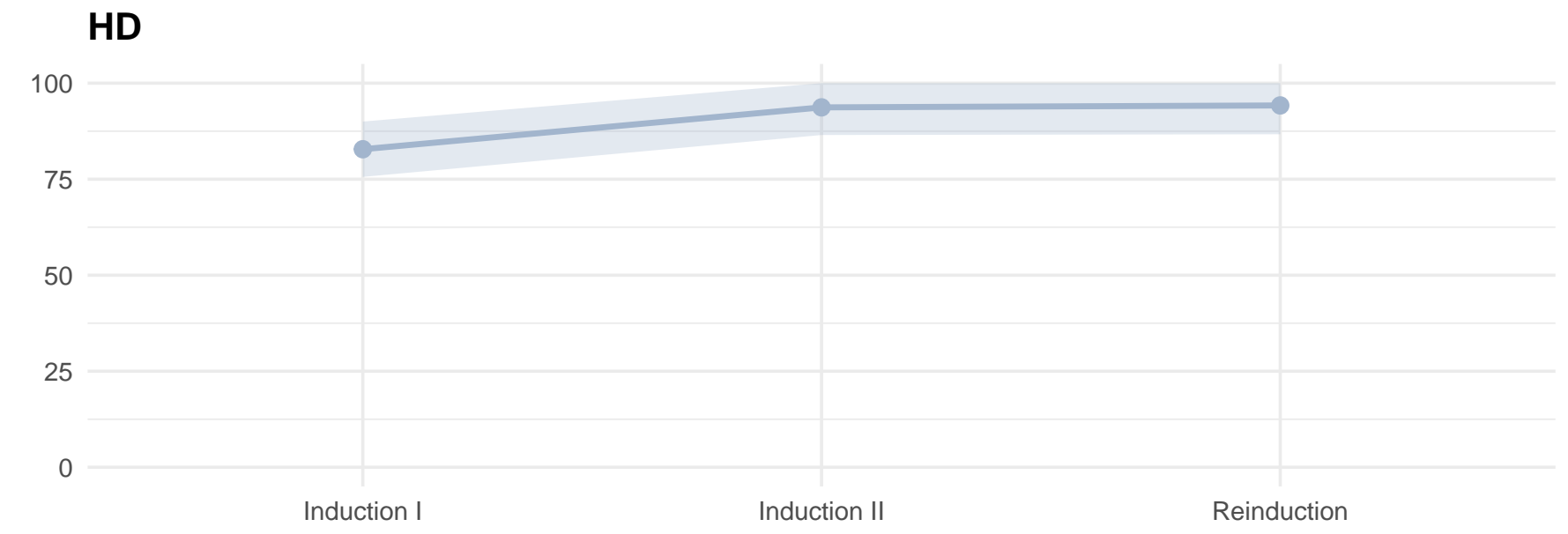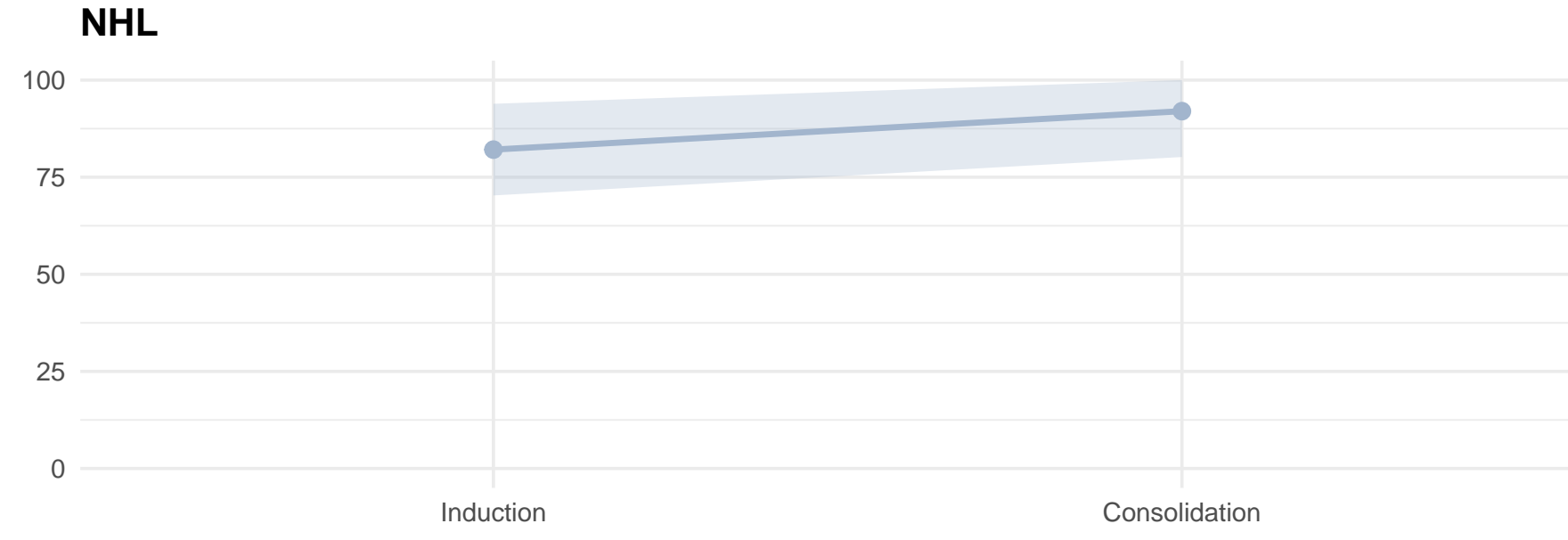

Supplement: Figure S4 [file mmc4.pdf]
