# Supplementary material for: Cancer-related fatigue in children during treatment: a 5-year cohort study of daily patient-reported outcomes with clinical implications
Source: eClinicalMedicine. 2025 Oct 30;90:103607. doi: 10.1016/j.eclinm.2025.103607 (PMC12613073; doi:10.1016/j.eclinm.2025.103607)
Supplement: Figure S2 [file mmc2.pdf]

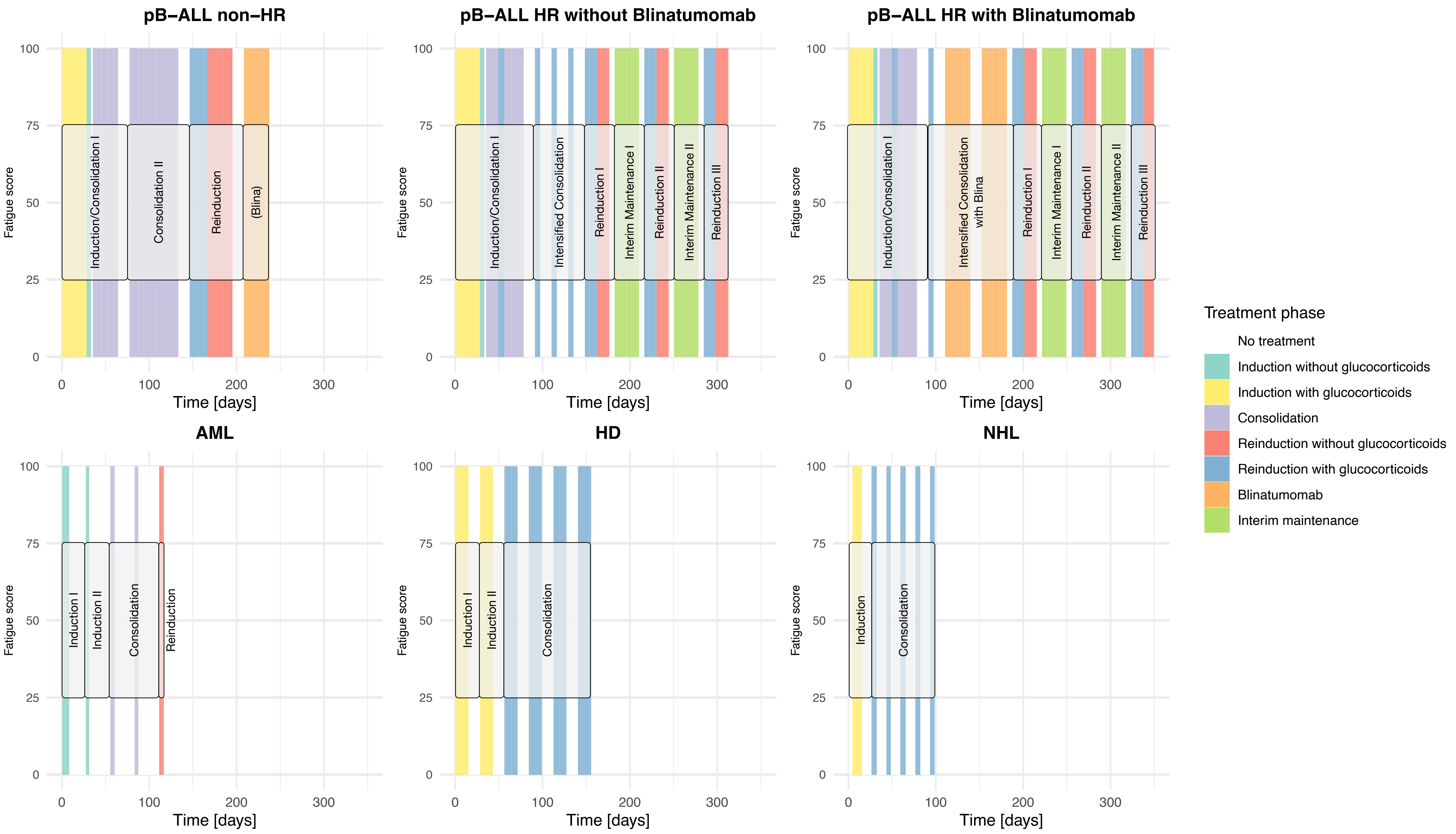

pB−ALL non−HR
pB−ALL HR without Blinatumomab
pB−ALL HR with Blinatumomab
AML
HD
NHL
Fatigue score
Time [days]
0
25
50
75
100
0
100
200
300
Induction/Consolidation I
Consolidation II
Reinduction
(Blina)
Intensified Consolidation
Reinduction I
Interim Maintenance I
Reinduction II
Interim Maintenance II
Reinduction III
Intensified Consolidation with Blina
Induction I
Induction II
Consolidation
Induction
Treatment phase
No treatment
Induction without glucocorticoids
Induction with glucocorticoids
Consolidation
Reinduction without glucocorticoids
Reinduction with glucocorticoids
Blinatumomab
Interim maintenance
