# Supplementary material for: Cancer-related fatigue in children during treatment: a 5-year cohort study of daily patient-reported outcomes with clinical implications
Source: eClinicalMedicine. 2025 Oct 30;90:103607. doi: 10.1016/j.eclinm.2025.103607 (PMC12613073; doi:10.1016/j.eclinm.2025.103607)
Supplement: Figure S3 [file mmc3.pdf]

pB-ALL non-HR, AML, HD, NHL

$$y_{ij} = \beta_0 + \sum_{k=2}^{K} \beta_k \, D_{ijk} + b_i + \varepsilon_{ij}$$

y_{ij}	Median fatigue for patient i at measurement j
β_0	Intercept (mean fatigue at the reference treatment block)
β_k	Fixed-effect coefficient for treatment block $k = 2, \ldots, K$
D_{ijk}	Indicator variable: 1 if measurement j of patient i is in block k, 0 otherwise
b_i	Patient-specific random intercept, $b_i \sim N(0, \tau^2)$
ε_{ij}	Residual error for patient i at measurement j, with AR(1) correlation across measurements
τ^2	Variance of the random intercepts

pB-ALL HR

$$y_{ij} = \beta_0 + \sum_{k=2}^{K} \beta_k \, D_{ijk} + \gamma \, B_{ij} + b_i + \varepsilon_{ij}$$

y_{ij}	Median fatigue for patient i at measurement j
β_0	Intercept (median fatigue at the reference treatment block)
β_k	Fixed-effect coefficient for treatment block $k = 2, \ldots, K$
D_{ijk}	Indicator variable: 1 if measurement j of patient i is in block k, 0 otherwise
γ	Fixed-effect coefficient for receiving Blinatumomab in the intensified consolidation block
B_{ij}	Indicator variable: 1 if patient i received Blinatumomab at measurement j, 0 otherwise
b_i	Patient-specific random intercept, $b_i \sim N(0, \tau^2)$
ε_{ij}	Residual error for patient i at measurement j, with AR(1) correlation across measurements
τ^2	Variance of the random intercepts
