# Supplementary material for: Cancer-related fatigue in children during treatment: a 5-year cohort study of daily patient-reported outcomes with clinical implications
Source: eClinicalMedicine. 2025 Oct 30;90:103607. doi: 10.1016/j.eclinm.2025.103607 (PMC12613073; doi:10.1016/j.eclinm.2025.103607)

Set missing values to 87.5

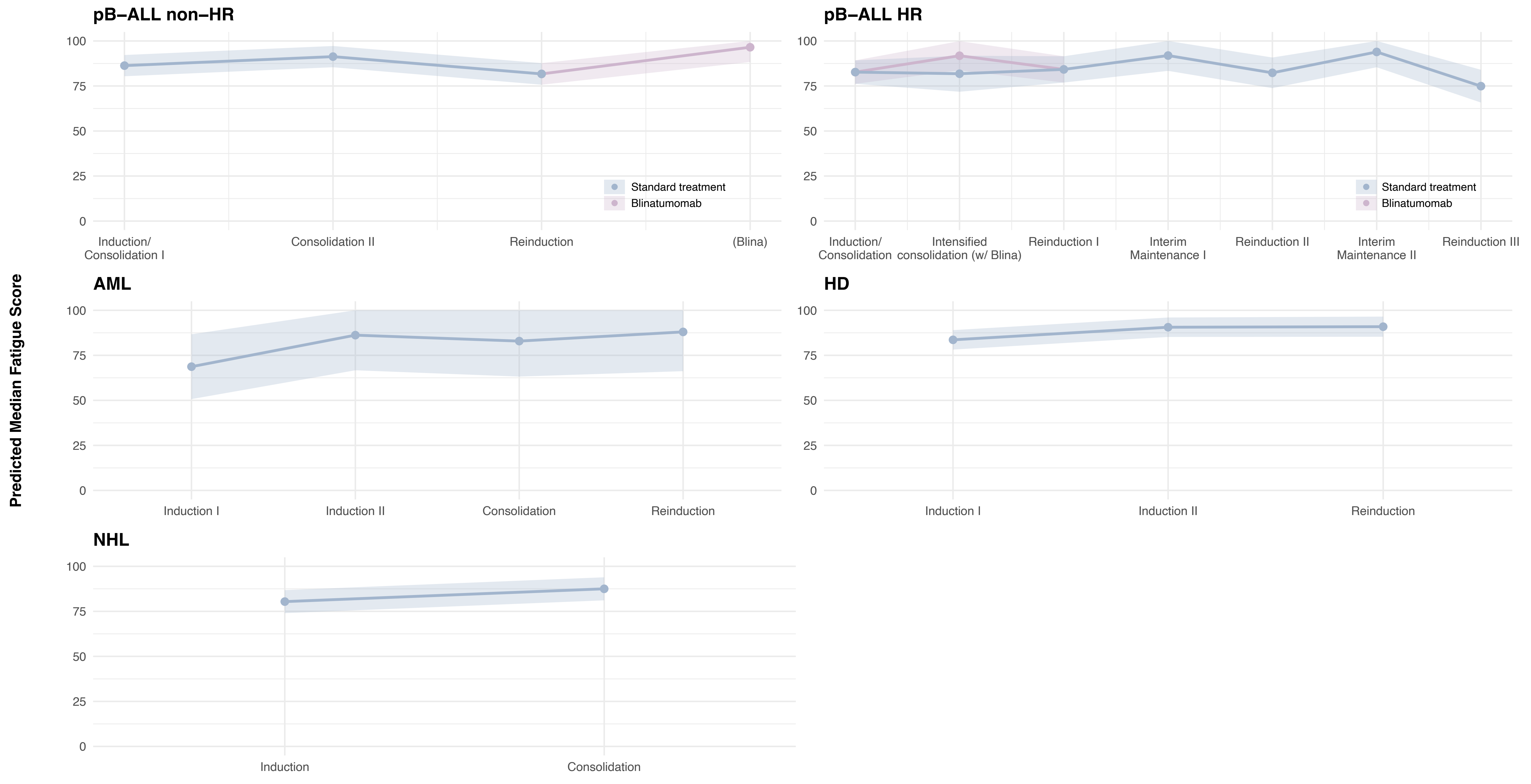

Impute missing values from random draws (with replacement) from each patient’s observed CRF distribution

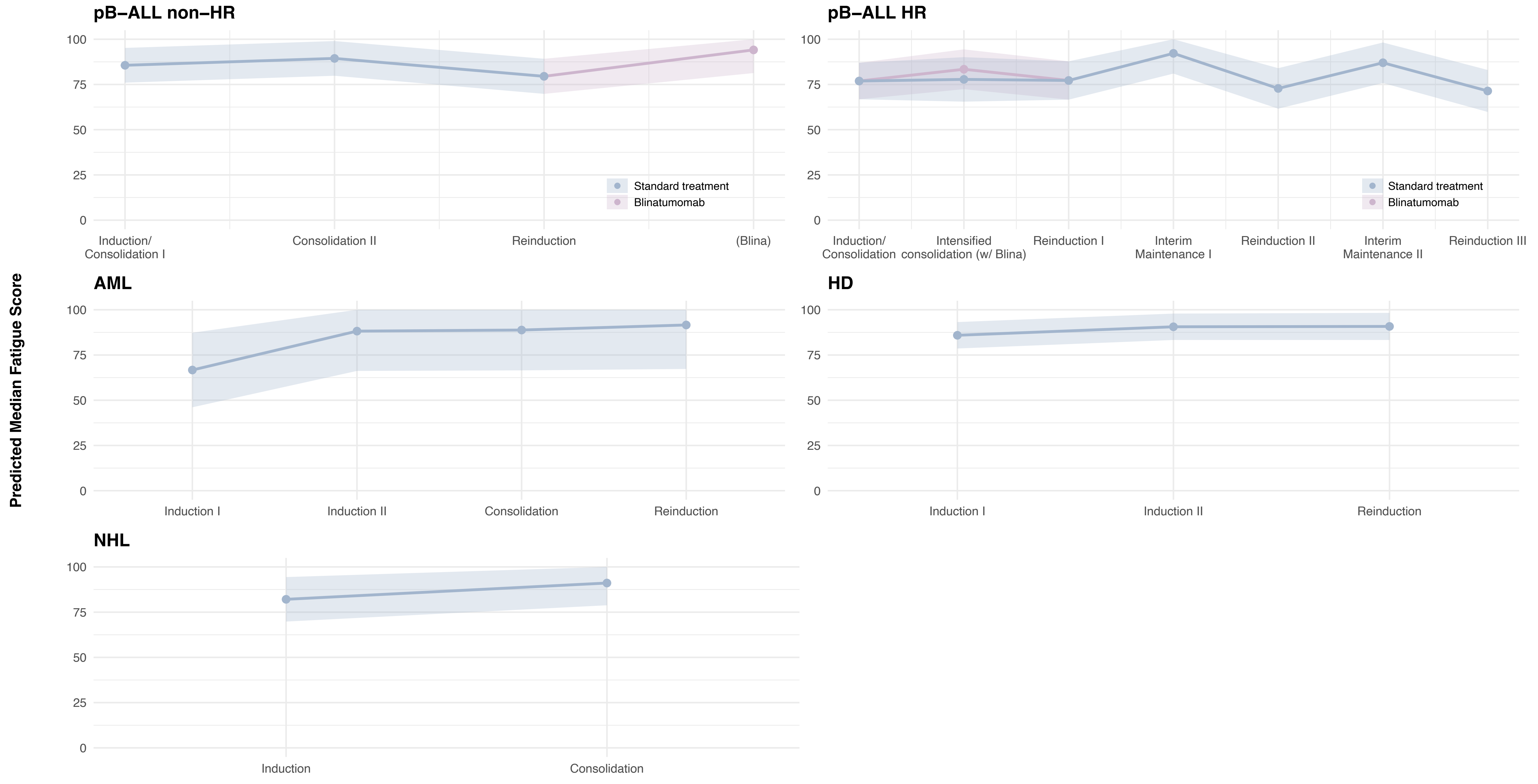

Impute missing values from random draws (with replacement) from each patient’s observed CRF distribution and add 12.5

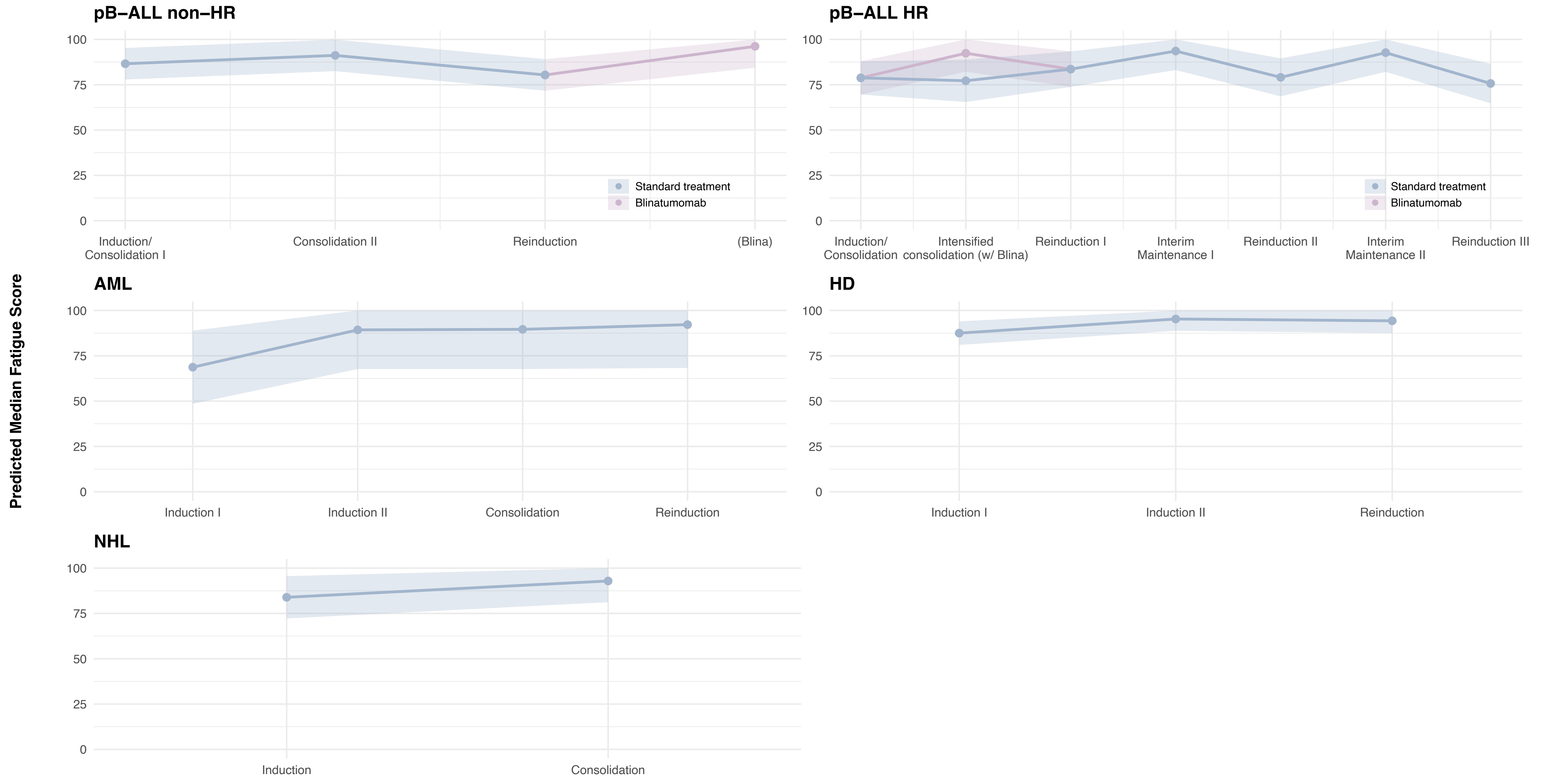

Supplement: Figure S5 [file mmc5.pdf]
