# Supplementary material for: Cancer-related fatigue in children during treatment: a 5-year cohort study of daily patient-reported outcomes with clinical implications
Source: eClinicalMedicine. 2025 Oct 30;90:103607. doi: 10.1016/j.eclinm.2025.103607 (PMC12613073; doi:10.1016/j.eclinm.2025.103607)
Supplement: Appendix [file mmc6.docx]

**Appendix**

Table of contents

[**Supplemental Figure 1 – Flow Chart** 2](#_Toc209024515)

[**Supplemental Figure 2 – Visualization of treatment phases for calculation** 3](#_Toc209024516)

[**Supplemental Figure 3 – Mixed-model equations** 4](#_Toc209024517)

[**Supplemental Figure 4 – Predicted median CRF with 95% confidence interval** 5](#_Toc209024518)

[**Supplemental Figure 5 – Sensitivity analysis** 6](#_Toc209024519)

[**Supplemental Table 1 - Demographics and clinical characteristics of excluded patients** 7](#_Toc209024520)

[**Supplemental Table 2 – Completion rate** 8](#_Toc209024521)

[**Supplemental Table 3 – CRF scores on response rate** 9](#_Toc209024522)

# **Supplemental Figure 1 – Flow Chart**


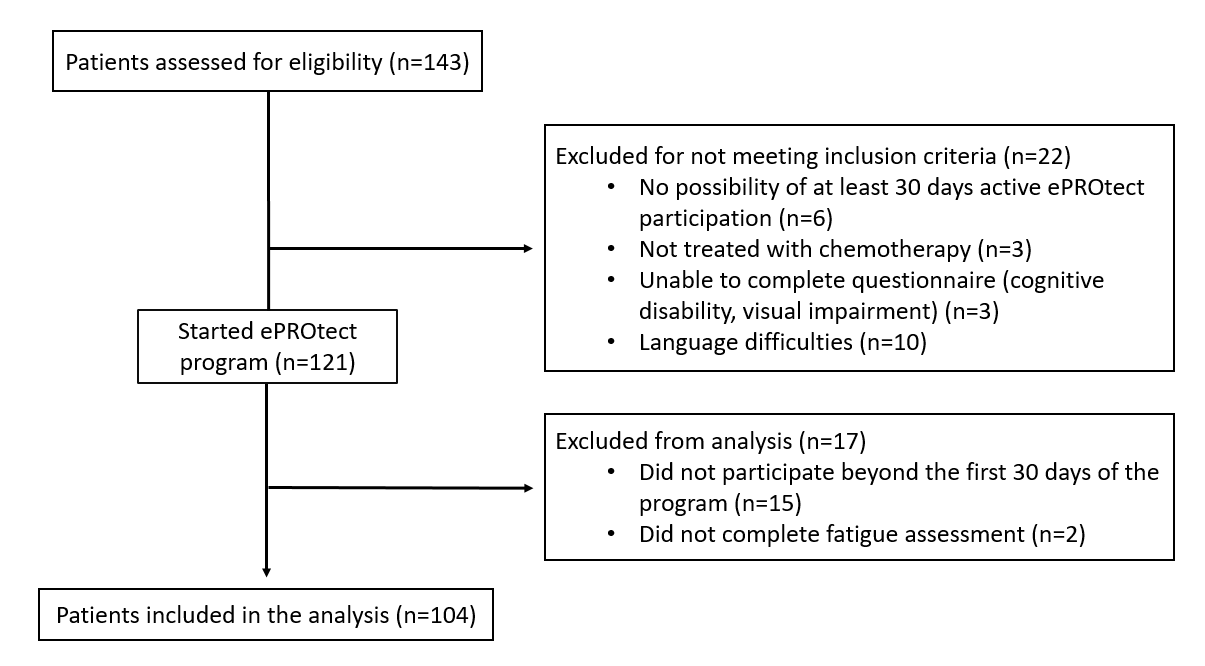


Between May 1, 2020 and December 31, 2024, all pediatric and adolescent oncology patients under the age of 18 were consecutively recruited. 22 patients were excluded for not meeting the inclusion criteria due to various reasons. A total of 121 patients were introduced to ePROtect, which allowed them or their care givers to complete clinical outcome assessments online on a daily basis. 17 patients were excluded from the analysis due to either participating for fewer than 30 days or not completing the fatigue specific questions

# **Supplemental Figure 2 – Visualization of treatment phases for calculation**


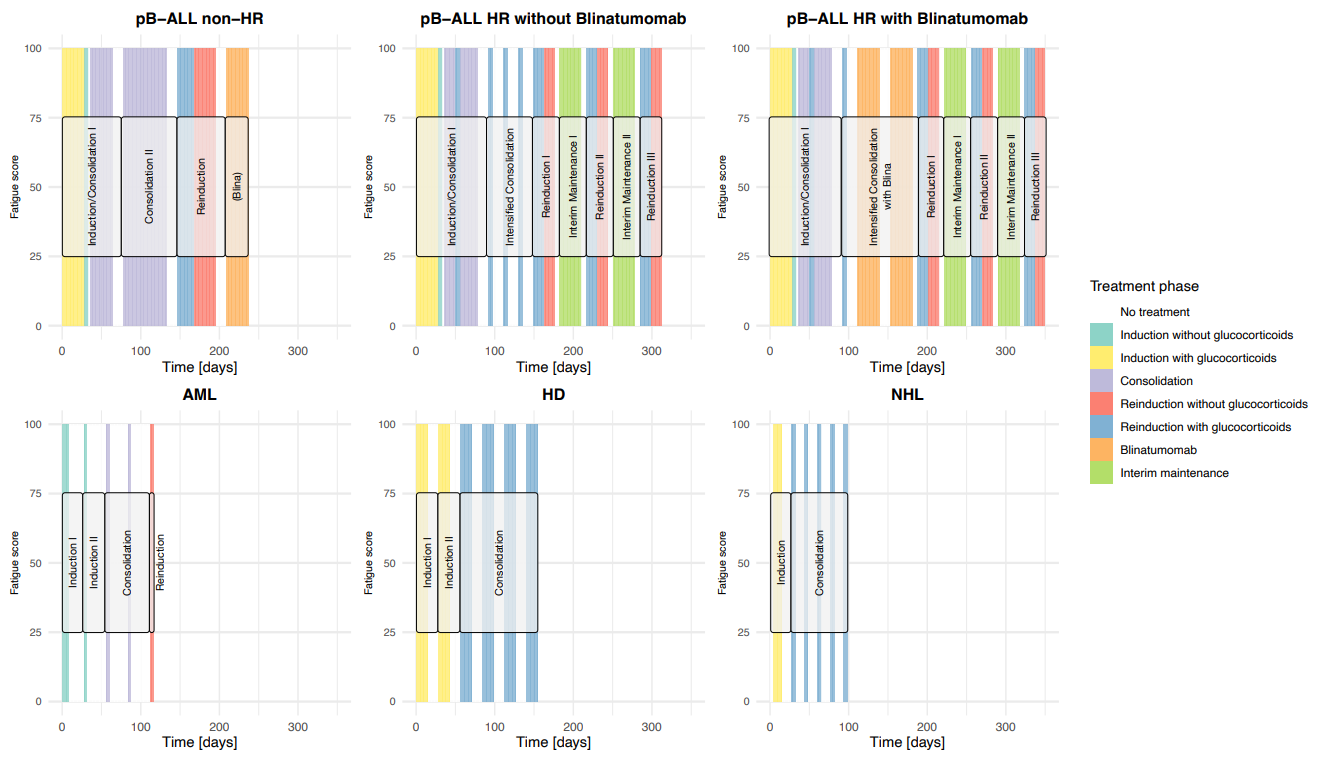


For statistical analysis, specific therapy elements were grouped according to drug properties, their side effect profiles, and clinical expertise, in order to improve comparability and account for potential delayed effects on Cancer-related fatigue.

Abbreviations: pB, pre B-cell; ALL, acute lymphoblastic leukemia; HR, high risk; AML, acute myeloid leukemia; HD, Hodgkin disease; NHL, Non-Hodgkin lymphoma

# **Supplemental Figure 3 – Mixed-model equations**

pB-ALL non-HR, AML, HD, NHL:


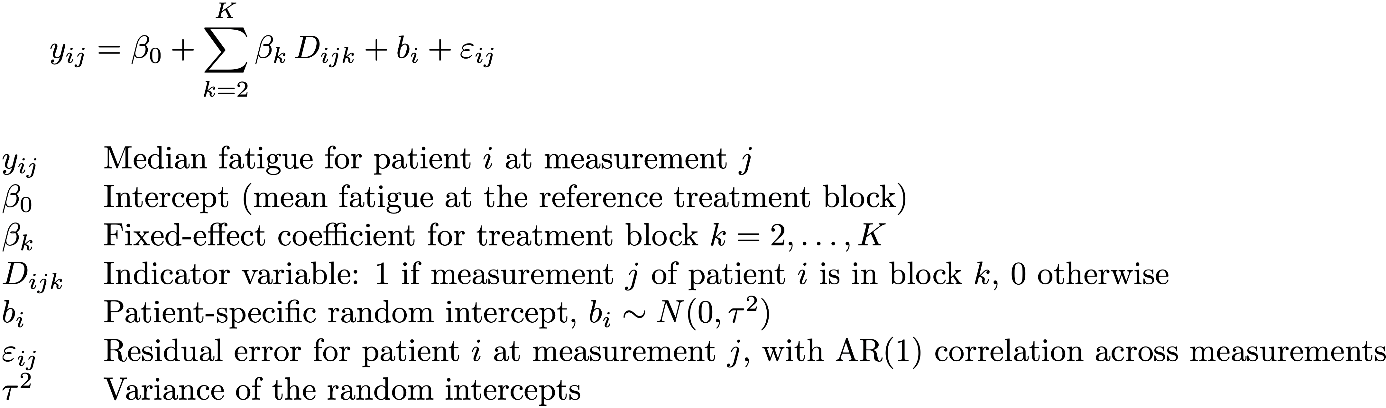


pB-ALL HR:


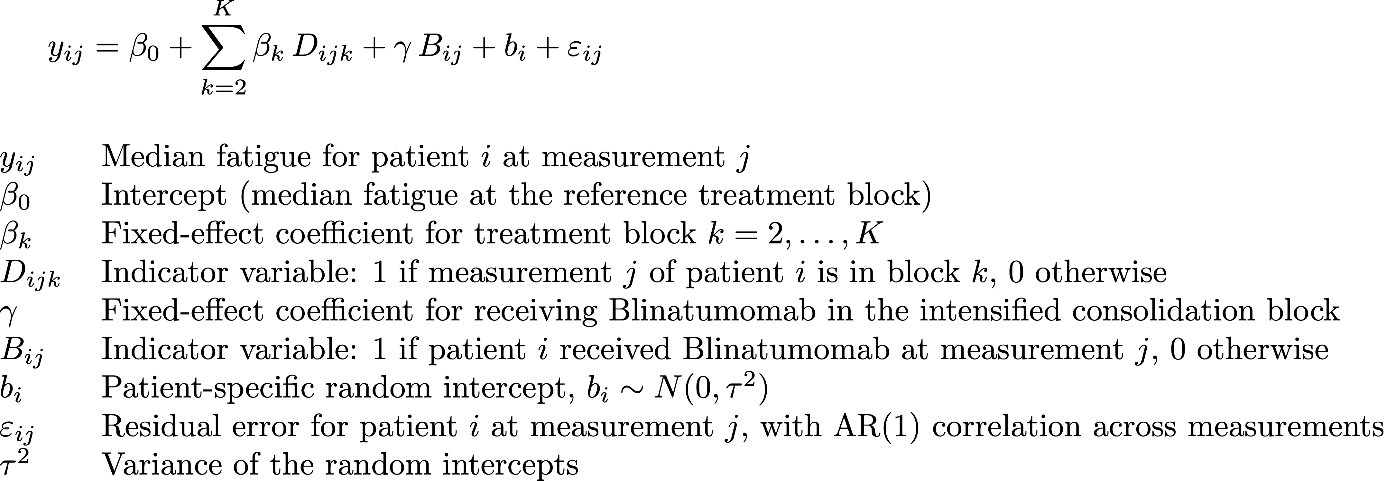


Abbreviations: pB, pre B-cell; ALL, acute lymphoblastic leukemia; HR, high risk; AML, acute myeloid leukemia; HD, Hodgkin disease; NHL, Non-Hodgkin lymphoma

# **Supplemental Figure 4 – Predicted median CRF with 95% confidence interval**


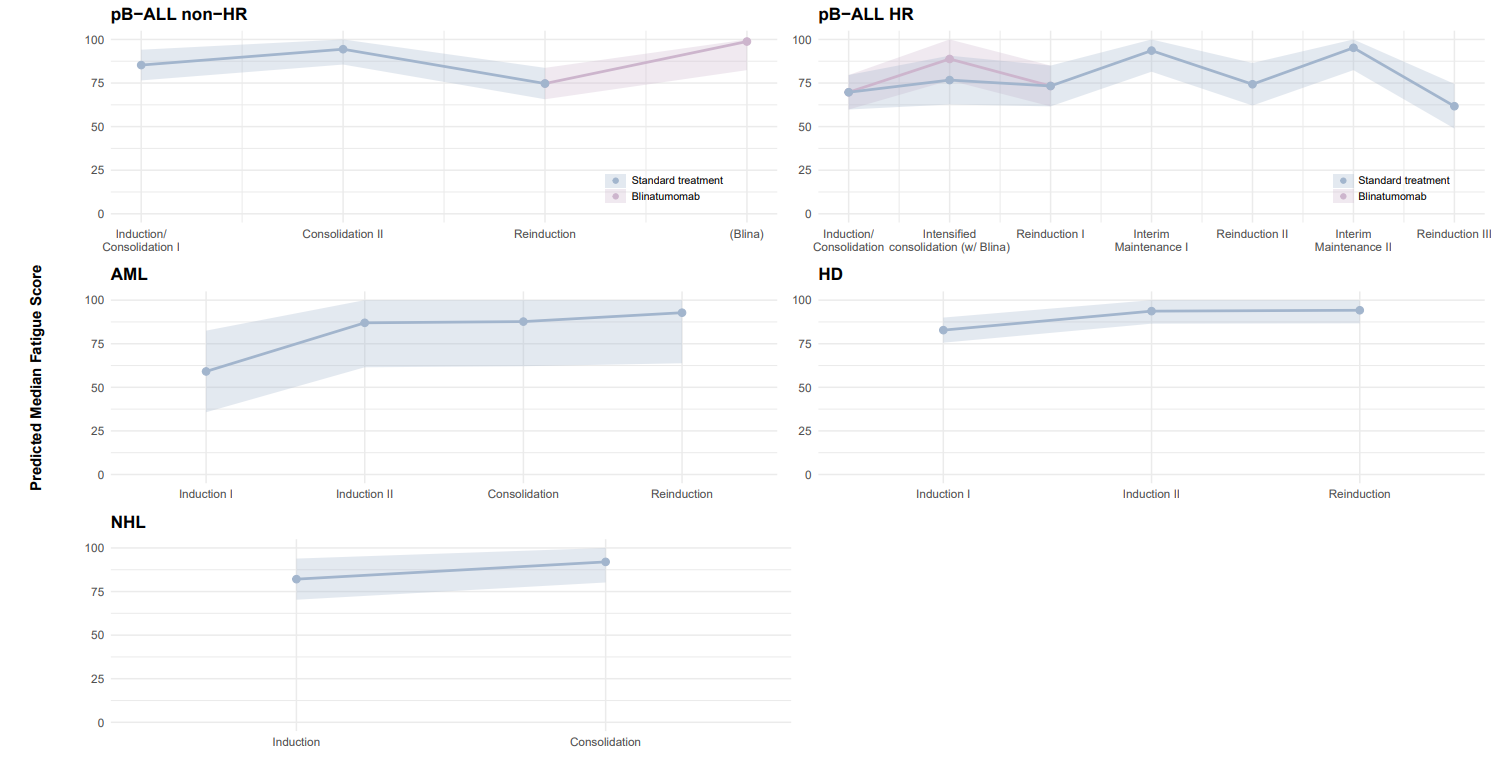


Progression of median cancer-related fatigue (CRF) across therapy blocks is displayed in different diagnoses and treatments. Purple indicates values of patients receiving Blinatumomab in that block.

Abbreviations: pB, pre B-cell; ALL, acute lymphoblastic leukemia; HR, high risk; AML, acute myeloid leukemia; HD, Hodgkin disease; NHL, Non-Hodgkin lymphoma

# **Supplemental Figure 5 – Sensitivity analysis**


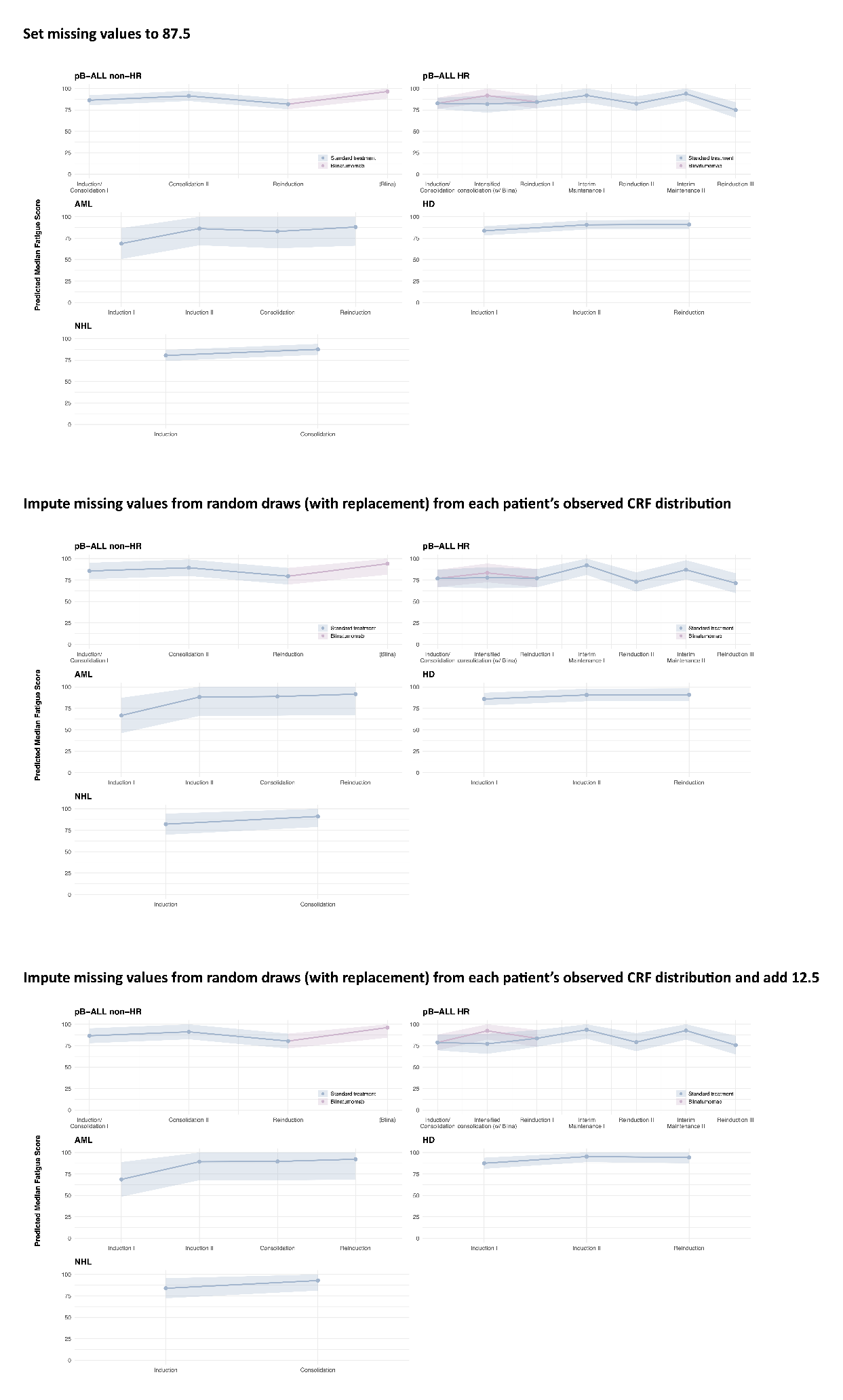


Abbreviations: pB, pre B-cell; ALL, acute lymphoblastic leukemia; HR, high risk; AML, acute myeloid leukemia; HD, Hodgkin disease; NHL, Non-Hodgkin lymphoma; CRF, cancer-related fatigue

#

# **Supplemental Table 1 - Demographics and clinical characteristics of excluded patients**

| **Characteristic** | **Excluded patients (N = 39) ^a^** |
| --- | --- |
| Age, median (IQR), y | 9·8 (2·8, 13·4) |
| Sex  Male  Female | 26 (64·1)  14 (35·9) |
| Underlying diagnosis  ALL  AML  HD  NHL  CNS tumor  Others | 5 (12·8)  1 (2·6)  7 (17·9)  3 (7·7)  7 (17·9)  16 (41·0) |

^a^ Unless indicated otherwise, data are expressed as No. (%) of patients.

Abbreviations: y, years; ALL, acute lymphoblastic leukemia; AML, acute myeloid leukemia; HD, Hodgkin disease; NHL, Non-Hodgkin lymphoma; CNS, central nervous system.

# **Supplemental Table 2 – Completion rate**

| **Characteristic** | **Completion rate (IQR)** |
| --- | --- |
| Overall | 56·1% (30·3, 76·2) |
| Age group  0-4 y  5-7 y  8-18 y | 55·5% (35·9, 82·2)  59·2% (30·0, 72·4)  55·6% (30·1, 72·4) |
| Sex  Male  Female | 47·7% (24·4, 76·9)  60·3% (46·2, 74·3) |
| Diagnosis group  ALL (HR)  ALL (Non-HR)  AML  Hodgkin  NHL  CNS  Other | 39·6% (22·3, 69·5)  66·7% (29·2, 83·6)  46·3% (38·4, 58·3)  59·3% (33·2, 80.8)  57·8% (28·9, 67·4)  56·8% (47·3, 80·7)  58·2% (29·8, 73·2) |

Abbreviations: y, years; ALL, acute lymphoblastic leukemia; HR high risk; AML, acute myeloid leukemia; NHL, non-Hodgkin lymphoma; CNS, central nervous system.

# **Supplemental Table 3 – CRF scores on response rate**

|  | **Unadjusted**  β and 95% CI | **Adjusted^a^**  β and 95% CI |
| --- | --- | --- |
| Response rate | 7.2 (-6.0, 20.4) | 2.5 (-10.2, 15.1) |
| Low responder  High responder^b^ | Ref.  2.3 (-4.9, 9.5) | Ref.  -0.2 (-7.2, 6.8) |

^a^ adjusted for sex, age, and diagnosis group

^b^ response rate at or above the cohort median

Abbreviations: *CI,* confidence interval; *Ref.,* referent
